# Selective Separation of Inorganic and Organic Carbonates in Aqueous Solutions by Reverse Osmosis (RO) and Nanofiltration (NF) Membranes

**DOI:** 10.3390/membranes16070248

**Published:** 2026-07-20

**Authors:** Rahma Al Busaidi, Budoor Al Umairi, Zulfiqar Ahmad Rehan, Mohammed Al-Abri

**Affiliations:** 1 Department of Physics, College of Science, Sultan Qaboos University, Al-Khoud, P.O. Box 33, Muscat 123, Oman; 2 Nanotechnology Research Center, Sultan Qaboos University, Al-Khoud, P.O. Box 33, Muscat 123, Oman; 3 Department of Chemistry, College of Science, Sultan Qaboos University, Al-Khoud, P.O. Box 33, Muscat 123, Oman; bsss@squ.edu.om (B.A.U.); z.rehan@squ.edu.om (Z.A.R.); 4 Department of Petroleum and Chemical Engineering, Sultan Qaboos University, Al-Khoud, P.O. Box 33, Muscat 123, Oman

**Keywords:** inorganic carbonates, organic carbonates, carbonate separation, reverse osmosis, nanofiltration, zeta potential, molecular weight cut-off

## Abstract

This study presents a systematic comparison of commercial reverse osmosis (RO) and nanofiltration (NF) membranes for the separation of inorganic and organic carbonates from aqueous solutions, providing insight into the roles of membrane pore structure and surface charge in governing separation mechanisms. Membrane molecular weight cut-off (MWCO), zeta potential, and thermal stability were characterized and correlated with separation performance. The RO membrane exhibited a lower MWCO and a more negative surface charge than the NF membrane, resulting in superior rejection of both inorganic and organic carbonates. For inorganic carbonates, rejection increased with pH owing to enhanced carbonate ionization and stronger electrostatic repulsion, reaching 91–98% for the RO membrane compared with 60–95% for the NF membrane. In contrast, the rejection of neutral organic carbonates was governed primarily by steric exclusion, with the RO membrane achieving approximately 80% rejection, whereas the NF membrane exhibited negligible removal. These findings demonstrate the combined influence of membrane pore size and surface charge on carbonate separation and provide practical guidance for selecting commercial membranes for efficient carbonate removal in water treatment applications.

## 1. Introduction

Access to clean and safe water is one of the most pressing global challenges, driven by rapid population growth, industrial expansion, and increasing environmental pollution [1,2,3]. In response, membrane-based technologies have emerged as highly efficient and versatile tools for advanced water and wastewater treatment [4]. Among these, reverse osmosis (RO) and nanofiltration (NF) membranes are widely used for the removal of a broad spectrum of contaminants, ranging from dissolved salts and organic compounds to pathogens and heavy metals [5]. These technologies are particularly valued for their high rejection efficiency, modular design, and low chemical usage compared to conventional treatment methods [6].

Beyond conventional polymeric RO and NF membranes, increasing attention has been directed toward two-dimensional (2D) nanomaterials for next-generation water purification. Among these, graphene oxide, MXenes, and their hybrid assemblies have emerged as promising membrane materials because of their hydrophilic surfaces, tunable interlayer spacing, abundant surface functional groups, and excellent mechanical stability. These characteristics enable improved water transport while maintaining high selectivity through the combined effects of size exclusion, electrostatic interactions, and engineered transport pathways. Recent advances in graphene oxide and MXene-based hybrid membranes further demonstrate the potential of integrating nanostructured materials with conventional membrane technologies to enhance permeability, antifouling properties, and separation efficiency [7]. Despite these developments, commercial polymeric RO and NF membranes remain the benchmark for large-scale water treatment, making systematic investigations of their separation performance and transport mechanisms essential for both current applications and the future design of advanced membrane systems.

The removal of carbonate species, specifically inorganic carbonate (CO_3_^2−^) and bicarbonate (HCO_3_^−^), as well as organic carbonates such as propylene carbonate and ethylene carbonate, has received relatively limited attention in membrane-based separation research. This is despite their growing relevance in both natural and industrial water systems. Carbonate and bicarbonate ions play a central role in water chemistry and scaling phenomena, while organic carbonates are increasingly detected in industrial effluents, particularly from chemical manufacturing, pharmaceutical production, lithium-ion battery electrolyte processing, and solvent recovery operations [8]. Although organic carbonates are generally considered to have low acute toxicity, their persistence and accumulation in aquatic environments raise concerns regarding long-term ecological impacts. Furthermore, the high solubility, low molecular weight, and, in some cases, neutral charge of these compounds make their removal challenging for conventional water treatment processes [9]. Consequently, there is a clear need for systematic evaluation of reverse osmosis (RO) and nanofiltration (NF) membrane performance in rejecting both inorganic and organic carbonate species to ensure effective treatment and compliance with increasingly stringent water quality regulations.

Comprehensive studies addressing the simultaneous separation of inorganic and organic carbonates using membrane filtration remain scarce. Most membrane separation studies have focused on common ions such as sulfate, chloride, and nitrate or on neutral organics such as pharmaceuticals and endocrine-disrupting compounds [10,11]. Carbonate species, due to their dual chemical nature, buffering behavior, and interactions with co-ions and membrane surfaces, present a unique challenge and require dedicated investigation. Furthermore, parameters such as solution pH, ionic strength, membrane surface charge, and hydrophilicity all play crucial roles in determining the transport and rejection behavior of carbonate compounds through dense polymeric membranes [12].

Although the rejection performance of commercial RO and NF membranes has been widely investigated for conventional inorganic salts, a systematic comparison of their ability to separate both charged inorganic carbonates and neutral organic carbonates under identical operating conditions remains limited. Moreover, the influence of membrane physicochemical properties, including molecular weight cut-off (MWCO), surface charge, and thermal characteristics, on the rejection behavior of ionic and neutral carbonate species by reverse osmosis (RO) and nanofiltration (NF) membranes has not been comprehensively elucidated. Addressing this knowledge gap is essential for understanding the separation behavior of carbonate species and for guiding the selection of appropriate commercial RO and NF membranes for carbonate-containing streams in water treatment and industrial applications. Therefore, this study systematically compares the performance of commercial RO and NF membranes for the removal of inorganic and organic carbonates while correlating membrane characteristics with separation behavior to provide mechanistic insight and practical guidance for membrane selection.

This study seeks to address the limited understanding of carbonate removal from water in membrane processes by systematically investigating the separation of both inorganic and organic carbonates using commercial reverse osmosis (RO) and nanofiltration (NF) membranes. Special emphasis is placed on elucidating the mechanisms governing rejection, particularly the influence of membrane surface charge, pore size, and carbonate speciation across varying pH levels. Key findings reveal that RO membranes consistently outperform NF membranes, achieving up to 98% rejection of inorganic carbonates and approximately 80% removal of neutral organic carbonates, while NF membranes show moderate rejection of inorganic carbonates and negligible removal of organic carbonates. These results highlight the critical role of charge repulsion and membrane structure in determining separation efficiency. The insights gained contribute to a deeper mechanistic understanding of membrane-based separation and support the advancement of sustainable water treatment technologies aligned with global water security and environmental protection objectives.

## 2. Materials and Methods

### 2.1. Materials

Commercial reverse osmosis (RO) and nanofiltration (NF) membranes were employed in this study. The RO membrane was manufactured by Toray Industries, Inc. (Tokyo, Japan), and the nanofiltration (NF) membrane was manufactured by DuPont Water Solutions (formerly DOW FilmTec, Edina, MN, USA). Both membranes were purchased from Sterlitech Corporation (Auburn, WA, USA). The key technical specifications of the commercial RO and NF membranes, including the manufacturer’s data sheets and nominal membrane properties, are provided in the Supplementary Materials Section S1 [13,14].

All chemicals used were of analytical grade. Inorganic carbonate salts, including ammonium bicarbonate (NH_4_HCO_3_), potassium bicarbonate (KHCO_3_), sodium bicarbonate (NaHCO_3_), ammonium carbonate ((NH_4_)_2_CO_3_), and sodium carbonate (Na_2_CO_3_), as well as the organic carbonate compounds ethylene carbonate (C_3_H_6_O_3_) and propylene carbonate (C_4_H_6_O_3_), were obtained from Sigma-Aldrich (St. Louis, MO, USA). All aqueous solutions were prepared using deionized (DI) water. Membrane filtration experiments were conducted using an HP4750 stirred dead-end filtration cell (Sterlitech Corporation, Auburn, WA, USA) to evaluate membrane separation performance.

### 2.2. Method

#### 2.2.1. Physicochemical Characterization of RO and NF Membranes

To evaluate the physicochemical properties and separation characteristics of the membranes, a series of characterization techniques were performed, including zeta potential analysis, molecular weight cut-off (MWCO) measurements, and thermogravimetric analysis (TGA).

##### Zeta Potential

The surface charge properties of the membranes were investigated using the streaming potential technique with a SurPASS 3 electrokinetic analyzer (Anton Paar GmbH, Graz, Austria). Membrane samples were cut into rectangular pieces (2 cm × 1 cm) and fixed onto the sample holders using double-sided adhesive tape. Measurements were conducted by placing two membrane samples inside the adjustable gap cell to form a flow channel with an effective capillary height of approximately 100 μm, which minimizes the contribution of the porous support layer and ensures reliable surface-sensitive measurements [15,16].

The electrolyte solution consisted of a mixture of 0.005 M LiCl and 0.005 M KCl prepared in deionized (DI) water. These electrolytes were selected due to their minimal interaction with membrane surfaces and negligible ion exchange effects during the relatively short measurement period (~30 s) [16]. The electrolyte solution was circulated through the membrane channel under an applied pressure range of 200–600 mbar, and the resulting streaming potential was automatically measured and recorded by the instrument software. The pH of the electrolyte was systematically adjusted using dilute 1 mM HCl and 1 mM NaOH solutions to evaluate the zeta potential as a function of pH. The zeta potential values were automatically calculated by the instrument based on the Helmholtz–Smoluchowski equation [17]. Zeta potential analysis provides important insight into membrane surface charge behavior and membrane–solute electrostatic interactions, which play a significant role in ion rejection and selective separation performance.

##### Molecular Weight Cut-Off (MWCO)

The molecular weight cut-off (MWCO) of the membranes was determined using polyethylene glycol (PEG) standards with molecular weights of 200, 600, 1500, 4000, 10,000, 20,000, 40,000, and 100,000 Da (Sigma-Aldrich, St. Louis, MO, USA). Each PEG solution was prepared at a concentration of 1 g L^−1^ in DI water. Filtration experiments were conducted using clean membranes under an applied pressure of 3.5 bar.

Feed and permeate samples were analyzed using a high-performance liquid chromatography (HPLC) system (1260 Infinity II, Agilent Technologies, Santa Clara, CA, USA) equipped with a C8 silica column and an evaporative light scattering detector (ELSD). An acetonitrile/water mixture was used as the mobile phase at a flow rate of 1 mL min^−1^. The molecular weight distribution profiles of PEG in the feed and permeate solutions were obtained from the chromatograms, and the rejection percentage for each PEG molecular weight was calculated. The MWCO was defined as the molecular weight corresponding to 90% solute rejection [18]. This analysis provides an estimation of the effective membrane pore size and selectivity characteristics.

##### Thermogravimetric Analysis (TGA)

The thermal stability and compositional characteristics of the membranes were evaluated using thermogravimetric analysis (TGA) with a TGA analyzer (SDT Q600, TA Instruments, New Castle, DE, USA). Membrane samples were heated from room temperature to 800 °C at a heating rate of 10 °C min^−1^ under a nitrogen atmosphere with a gas flow rate of 50 mL min^−1^. The resulting weight-loss profiles were analyzed to identify thermal decomposition stages, evaluate polymer stability, and assess structural integrity following membrane treatment or exposure to organic carbonate compounds.

#### 2.2.2. Membrane Filtration Setup

A dead-end filtration system (HP4750 Stirred Cell, Sterlitech Corporation, Auburn, WA, USA) [19,20] was employed to evaluate the separation performance of commercial reverse osmosis (RO) and nanofiltration (NF) membranes toward inorganic and organic carbonate species. Membrane discs were mounted inside the filtration cell with an effective membrane area exposed to filtration. All filtration experiments were conducted at a constant pressure of 20 bars and room temperature. Prior to each experiment, membranes were compacted and stabilized by discarding the first 2 mL of permeate. All filtration experiments were operated at a stirring speed of 400 rpm to reduce concentration polarization by promoting mixing adjacent to the membrane surface. Although stirring substantially decreases the concentration boundary layer thickness, concentration polarization cannot be completely eliminated in dead-end filtration, particularly for relatively concentrated (0.1 M) feed solutions. Therefore, the measured permeate flux and rejection represent the combined effects of intrinsic membrane separation and concentration polarization under the selected operating conditions.

The rejection performance toward inorganic carbonate species was evaluated by measuring the electrical conductivity of the feed and permeate solutions using a calibrated conductivity meter. A conductivity–concentration calibration curve was established using standard inorganic carbonate solutions to estimate carbonate concentrations. The rejection efficiency (R, %) was calculated according to Equation (1):

(1) $$R\left(\%\right)=\left(1-\frac{C_{p}}{C_{f}}\right)\times 100$$

where ***C_p_*** is the carbonate concentration in the permeate, and ***C_f_*** is the concentration in the feed [20,21].

The rejection of organic carbonate compounds was determined using gas chromatography (GC) [22]. Feed and permeate samples were collected and analyzed using an Agilent 7890A GC system (Agilent Technologies, Santa Clara, CA, USA) equipped with a flame ionization detector (FID) and a capillary column suitable for volatile organic compound analysis. Nitrogen was used as the carrier gas under controlled flow conditions. The rejection efficiency of organic carbonates was subsequently calculated using Equation (1).

The permeate water flux (***J***) was determined using Equation (2):

(2) $$J=\frac{V}{A\cdot t}$$

where ***J*** is the flux (L/h·m^2^), ***V*** is the permeate volume (L), ***A*** is the membrane area (m^2^), and ***t*** is the filtration time (h) [20,21].

## 3. Results and Discussion

### 3.1. Membrane Characterization

To enable accurate prediction of membrane performance, fundamental properties including surface charge and molecular weight cut-off (MWCO) of both NF and RO membranes were characterized, since these parameters play a critical role in controlling solute selectivity and transport behavior.

**Figure 1:**
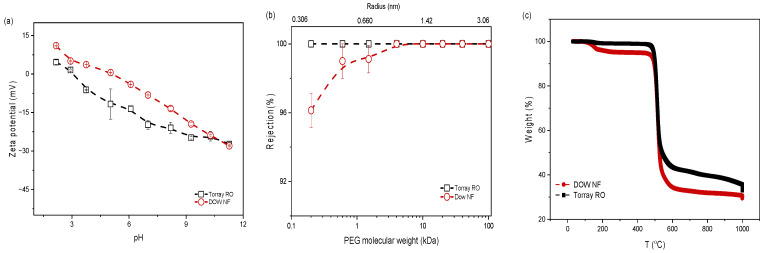
(**a**) Surface zeta potential of NF and RO membranes measured as a function of pH, showing increasing negativity with rising pH and RO exhibiting a more negative charge overall. (**b**) MWCO determination of NF and RO membranes measured using a mixture of eight different polyethylene glycol (PEG) molecules (200, 600, 1500, 4000, 10,000, 20,000, 40,000, and 100,000 Da) (Sigma-Aldrich), each in a concentration of 1 g L^−1^, which was filtered by dead-end filtration under 3.5 bar, illustrating NF’s partial rejection at low MW and full rejection above 4 kDa, while RO fully rejects all PEG sizes tested. The top x-axis shows PEG radius calculated as R(Å) = 0.262 (MW(g mole^−1^))0.5 − 0.3, as reported in Ref. [24]. (**c**) TGA analysis of NF and RO membrane active layers showing initial weight loss in NF starting at 160 °C with stability in RO up to 470 °C, followed by sharp decomposition of both membranes beyond 470 °C.

The surface zeta potential of both nanofiltration (NF) and reverse osmosis (RO) membranes was measured across a range of pH values to assess their surface charge behavior (Figure 1a). The RO membrane exhibited a more negative surface charge compared to the NF membrane throughout the pH spectrum. At acidic conditions (pH 2), the RO membrane showed a slightly positive zeta potential of +4.6 mV, which progressively decreased to a highly negative value of −27 mV at alkaline pH 11. In contrast, the NF membrane demonstrated a higher initial positive charge of +11 mV at pH 2, but its surface charge also became increasingly negative with rising pH, following a similar trend to the RO membrane at pH 11. This indicates that while both membranes gain negative charge as the environment becomes more alkaline [23], the RO membrane maintains a generally more negative surface potential, which can influence their separation performance and fouling characteristics differently.

The molecular weight cut-off (MWCO) of the NF and RO membranes was evaluated by filtering polyethylene glycol (PEG) standards of different molecular weights under a dead-end filtration configuration at an operating pressure of 3.5 bar (Figure 1b). The NF membrane demonstrated a rejection rate of approximately 96% for the smallest PEG tested, with a molecular weight of 0.2 kDa. This rejection progressively increased with the molecular weight of PEG, reaching complete rejection for PEG molecules of 4 kDa or larger. In contrast, the RO membrane exhibited full rejection of all PEG molecules across the entire range tested, indicating its tighter molecular sieving capabilities compared to the NF membrane [25,26].

Thermogravimetric analysis (TGA) was performed on the separated top selective layers of both NF and RO membranes to evaluate their thermal stability (Figure 1c). Prior to analysis, the active layers were carefully separated from the membrane support using adhesive tape to isolate the selective layer for accurate thermal characterization. The NF membrane exhibited an initial weight loss below 160 °C, which can be attributed to the evaporation of physically adsorbed water and residual moisture associated with hydrophilic surface groups [27,28]. A second gradual weight loss from approximately 160 to 470 °C reduced the membrane weight to about 93%, corresponding to the decomposition of oxygen-containing functional groups, such as carboxylic (–COOH), hydroxyl (–OH), and residual amide-related moieties present in the polyamide selective layer [27]. In contrast, the RO membrane showed only minor weight loss within this temperature range, indicating a lower concentration of thermally labile surface functionalities and a more highly crosslinked structure [28]. Above 470 °C, both membranes underwent a sharp and rapid weight loss due to the breakdown of the polyamide backbone and aromatic polymer chains, resulting in the formation of volatile decomposition products [28].

### 3.2. Carbonate Speciation and Membrane Charge Effects

**Figure 2:**
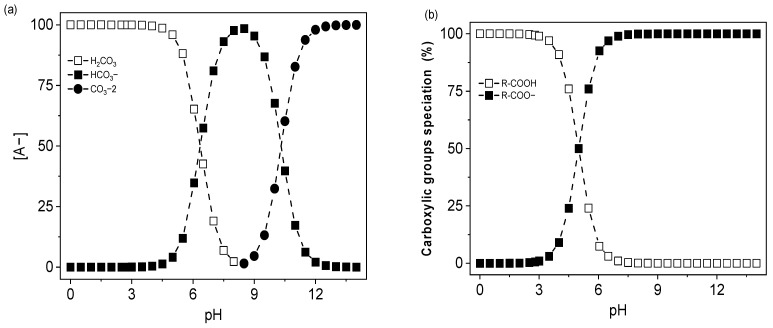
(**a**) Carbonate ionization and speciation across pH levels, described by the Henderson–Hasselbalch equation, reveal the stepwise transition from carbonic acid to bicarbonate and finally to carbonate ion. (**b**) Carboxylic acid ionization on the membrane surface above the pKa = 5.05, where the pKa value was taken from Ref. [31], leading to the formation of negatively charged carboxylate groups (–COO^−^) that enhance carbonate rejection through the Donnan exclusion effect.

The ionization behavior of carbonate can be quantitatively described and visualized using the Henderson–Hasselbalch equation, which relates the pH of a solution to the ratio of protonated to deprotonated species [29,30] (Figure 2a). By plotting pH against the logarithm of the ratio of acid to conjugate base for each dissociation step, the distribution of carbonate species across the pH scale can be effectively predicted. Further details regarding the carbonate speciation calculations and methodology are provided in Section S2 of the Supplementary Materials. This method clearly illustrates that at low pH, carbonic acid (H_2_CO_3_) is the dominant species; near neutral to slightly basic pH, bicarbonate ion (HCO_3_^−^) predominates; and at higher pH values, carbonate ion (CO_3_^2−^) becomes the major form.

Carbonate ionization occurs through a two-step deprotonation process characterized by pKa values of approximately 6.3 and 10.3. Initially, at low pH, the carbonate system mainly exists as neutral H_2_CO_3_. When the pH exceeds 6.3, H_2_CO_3_ loses a proton and converts to bicarbonate (HCO_3_^−^). By around pH 8, H_2_CO_3_ is virtually absent, and bicarbonate is the predominant species. As the pH rises above the second pKa of 10.3, bicarbonate undergoes further deprotonation to form the carbonate ion (CO_3_^2−^). Full ionization is achieved at pH values well above 10.3, where the carbonate ion is the dominant, fully deprotonated species in solution.

**Figure 3:**
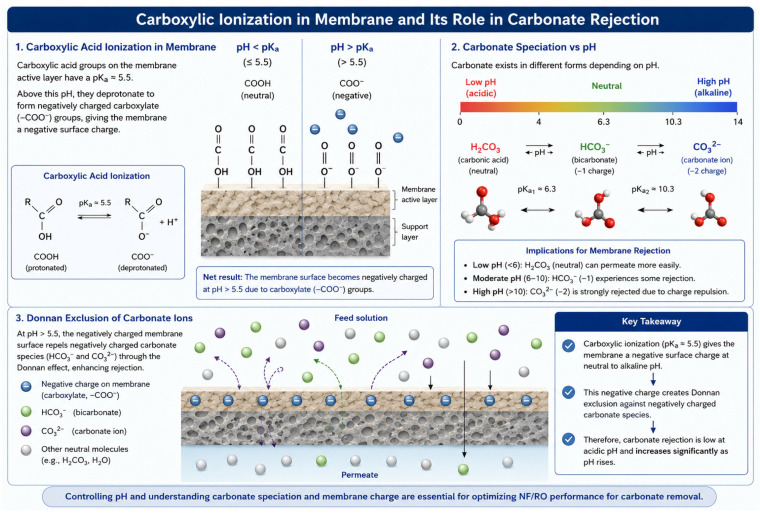
Schematic illustration of carboxylic acid ionization on the membrane surface and its influence on carbonate rejection by NF and RO membranes. Above the reported pKa value of carboxylic acid groups (~5.5; pKa value from Ref. [31] deprotonation generates negatively charged carboxylate groups (–COO^−^), which enhance the electrostatic repulsion of bicarbonate (HCO_3_^−^) and carbonate (CO_3_^2−^) ions through the Donnan exclusion effect, thereby increasing carbonate rejection at higher pH. This figure is an original illustration created by the authors. Artificial intelligence (AI) was used solely to assist in the graphical design of the figure, while the scientific conceptualization, interpretation, and verification of accuracy were performed entirely by the authors.

Understanding the carbonate ionization behavior and its speciation at different pH levels helps predict how carbonate species interact with nanofiltration (NF) and reverse osmosis (RO) membranes. Since NF and RO membranes reject ions based on both charge and size exclusion mechanisms, identifying the dominant carbonate species, whether neutral carbonic acid (H_2_CO_3_), bicarbonate (HCO_3_^−^), or carbonate ion (CO_3_^2−^), at a given pH enables estimation of membrane rejection efficiency. At higher pH values, where negatively charged bicarbonate and carbonate ions dominate, enhanced rejection is expected due to increased electrostatic repulsion between the ions and the negatively charged membrane surface. In contrast, at lower pH, neutral carbonic acid can permeate more easily through the membrane. The negative surface charge of NF and RO membranes mainly originates from the ionization of carboxylic acid functional groups present on the membrane active layer. Since carboxylic acid groups possess a pKa of approximately 5.05–5.5, deprotonation occurs at pH values above this range, resulting in the formation of negatively charged carboxylate groups (–COO^−^) (Figure 2b). This enhances the membrane surface negativity and promotes the Donnan exclusion effect, where negatively charged carbonate species are electrostatically repelled from the membrane surface, thereby improving carbonate rejection performance. Figure 3 summarizes the rejection mechanisms of carbonate.

### 3.3. Membrane Performance

The performance of the RO and NF membranes for carbonate removal was evaluated using a dead-end filtration system operated at a constant transmembrane pressure of 20 bar and ambient temperature (~25 °C). Five carbonate salts were investigated, namely ammonium bicarbonate (NH_4_HCO_3_), potassium bicarbonate (KHCO_3_), sodium bicarbonate (NaHCO_3_), ammonium carbonate ((NH_4_)_2_CO_3_), and sodium carbonate (Na_2_CO_3_), each prepared at a concentration of 0.1 M. The corresponding solution pH values were 7.86, 8.34, 8.86, 9.02, and 11.18, respectively, while their molecular weights were 79.06, 100.12, 84.01, 96.09, and 105.99 g mol^−1^.

The theoretical osmotic pressures of the feed solutions, estimated using the van’t Hoff equation [32,33] were approximately 4.9 bar for NH_4_HCO_3_, KHCO_3_, and NaHCO_3_ and 7.4 bar for (NH_4_)_2_CO_3_ and Na_2_CO_3_. More details about osmotic pressure calculation are provided in Section S3 of the Supplementary Materials. Since the applied filtration pressure of 20 bar was substantially higher than the osmotic pressure of all feed solutions, a positive net driving force was maintained throughout the experiments, ensuring effective permeation and minimizing the influence of osmotic pressure on membrane performance.

**Figure 4:**
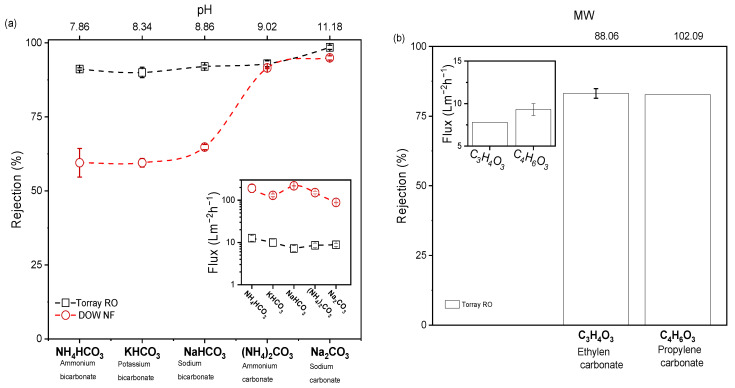
(**a**) RO and NF membrane performance in removing various carbonates from water was tested at different pH levels, showing increased rejection with higher pH and notable differences in flux between the two membranes. (**b**) RO membranes effectively reject ~80% of ethylene and propylene carbonate, while NF membranes show poor stability and no rejection.

The concentrations of inorganic carbonate species in both feed and permeate solutions were determined from conductivity measurements using concentration–conductivity calibration curves established separately for each carbonate salt. These calibration curves enabled accurate quantification of carbonate concentrations and subsequent calculation of membrane rejection efficiencies. Both membranes exhibited enhanced carbonate rejection with increasing solution pH, (Figure 4a). This behavior can be attributed to the pH-dependent ionization of carbonate species and the increasingly negative surface charge of the membrane. At lower pH, bicarbonate ions (HCO_3_^−^) are the dominant carbonate species, whereas at higher pH, particularly for sodium carbonate solutions, divalent carbonate ions (CO_3_^2−^) become predominant. Simultaneously, deprotonation of membrane carboxylic groups increases the membrane’s negative surface charge. The combined effect strengthens electrostatic repulsion between the negatively charged membrane and carbonate species, resulting in enhanced Donnan exclusion and higher rejection. Consequently, the RO membrane achieved rejection values of 91–92% for most carbonate salts, increasing to 98% for sodium carbonate. Similarly, the NF membrane exhibited rejection values of approximately 60% for bicarbonate salts, which increased significantly to 91% and 95% for ammonium carbonate and sodium carbonate, respectively.

The permeate flux decreased with increasing solution pH for both membranes, as shown in the inset of Figure 4a. For the RO membrane, the flux decreased from approximately 12 L m^−2^ h^−1^ for ammonium bicarbonate to 8 L m^−2^ h^−1^ for ammonium and sodium carbonate solutions. A similar trend was observed for the NF membrane, where the flux declined from 190 to 88 L m^−2^ h^−1^. This reduction can be attributed to the higher concentration of multivalent carbonate ions at elevated pH, which increases the osmotic pressure difference across the membrane and reduces the effective driving force for water transport. Furthermore, stronger electrostatic interactions between the membrane surface and highly charged carbonate species may promote concentration polarization near the membrane surface, further hindering water permeation. Although the theoretical osmotic pressures reported in this study were calculated from the bulk feed concentrations, the effective osmotic pressure opposing water transport is expected to be higher at the membrane surface because of concentration polarization. As carbonate rejection increases, particularly at higher pH where divalent carbonate species predominate, rejected ions accumulate within the concentration boundary layer adjacent to the membrane, increasing the local osmotic pressure and reducing the effective transmembrane pressure (ΔP − Δπ) [32,33]. Consequently, the observed decline in permeate flux at elevated pH is attributed to the combined effects of increased osmotic pressure and concentration polarization. Quantitative determination of the membrane-surface osmotic pressure would require direct concentration measurements or transport modeling and is therefore beyond the scope of the present study. Despite the flux reduction, the higher pH conditions resulted in substantially improved carbonate rejection, highlighting the important role of membrane charge and carbonate speciation in governing separation performance.

The removal of organic carbonates from water was investigated using 0.1 M solutions of ethylene carbonate (EC, 88.06 g mol^−1^) and propylene carbonate (PC, 102.09 g mol^−1^) as model compounds, (Figure 4b). The performance of nanofiltration (NF) and reverse osmosis (RO) membranes was evaluated in terms of rejection and permeate flux. The NF membrane exhibited negligible rejection toward both EC and PC, indicating that these neutral organic molecules readily permeated through the membrane. This behavior can be attributed to the absence of electrostatic repulsion and the relatively small molecular size of the carbonate molecules, which enables their transport through the larger effective pore structure of the NF membrane. In contrast, the RO membrane achieved significantly higher rejection values of approximately 80% for both compounds. The enhanced separation performance is primarily associated with the denser selective layer and smaller free-volume elements of the RO membrane, which impose stronger steric hindrance and diffusion resistance to the transport of organic carbonate molecules. Furthermore, the RO membrane maintained a stable water flux in the range of 7–9 L m^−2^ h^−1^, demonstrating effective separation without a substantial loss in permeability, as shown in the inset of Figure 4b. These findings highlight the limited suitability of NF membranes for the removal of neutral low-molecular-weight organic carbonates, whereas RO membranes offer a more effective approach for their separation from aqueous solutions through size-exclusion-controlled transport mechanisms.

The distinct separation behavior of the RO and NF membranes can be explained by their different mass transfer mechanisms. In RO membranes, water permeates through a dense, non-porous selective layer via the solution–diffusion mechanism, while solute transport is strongly restricted by the membrane’s low MWCO and highly negative surface charge. Consequently, both steric exclusion and electrostatic (Donnan) interactions contribute to the high rejection of inorganic carbonate ions, whereas neutral organic carbonates are rejected primarily through size exclusion. In contrast, NF membranes possess a looser selective structure with a higher MWCO, allowing greater solute transport through a combination of solution–diffusion and pore-flow mechanisms. As a result, the rejection of charged carbonate species depends on both electrostatic interactions and steric hindrance, while the larger pore structure permits significant passage of neutral organic carbonates.

## 4. Comparison with the Literature Review

Unlike previous studies that have focused exclusively on inorganic carbonate species, this work provides a systematic comparison of the separation behavior of both inorganic and organic carbonates using commercial RO and NF membranes under identical operating conditions. A comparison of the inorganic carbonate separation performance reported in the literature and in the present study is summarized in Table 1. Previous investigations demonstrated that nanofiltration membranes exhibit significantly higher rejection toward carbonate ions (CO_3_^2−^) than bicarbonate ions (HCO_3_^−^), primarily due to the stronger electrostatic repulsion experienced by the divalent carbonate species. Rinberg et al. reported that the NF270 membrane achieved approximately 40% rejection of bicarbonate ions and 80–90% rejection of carbonate ions, highlighting the role of Donnan exclusion in enhancing carbonate separation [34]. Similarly, Zhu et al. observed carbonate hardness rejections exceeding 97% and 80% for NF90 and NF270 membranes, respectively [35]. In the present study, the Toray RO membrane exhibited superior performance, achieving 91% bicarbonate rejection and 98% carbonate rejection, while the Dow NF membrane achieved 60% and 95% rejection of bicarbonate and carbonate ions, respectively. The higher rejection of carbonate ions observed for both membranes is attributed to the divalent nature of CO_3_^2−^, which results in stronger electrostatic repulsion from the negatively charged membrane surface compared with monovalent HCO_3_^−^. These findings are consistent with previous studies and further confirm that carbonate rejection increases substantially as carbonate species become more highly ionized and charge exclusion becomes the dominant separation mechanism.

## 5. Conclusions

This study provides a systematic comparison of commercial reverse osmosis (RO) and nanofiltration (NF) membranes for the separation of both inorganic carbonate ions and neutral organic carbonates, while correlating membrane physicochemical properties with separation behavior. The results demonstrate that membrane performance is governed by the combined effects of surface charge, pore structure, and carbonate speciation. As solution pH increased, the conversion of bicarbonate to divalent carbonate ions enhanced electrostatic exclusion, leading to rejection values of up to 98% for sodium carbonate by the RO membrane. In contrast, the rejection of neutral organic carbonates depended predominantly on steric hindrance, with the RO membrane achieving approximately 80% rejection, whereas the NF membrane exhibited negligible removal due to its larger effective pore size. The relationship established between membrane zeta potential, molecular weight cut-off (MWCO), and the rejection of both charged and uncharged carbonate species provides mechanistic insight into carbonate transport through pressure-driven membranes. These findings not only clarify the distinct separation mechanisms governing inorganic and organic carbonate removal but also provide practical guidance for selecting membrane technologies for carbonate-containing water streams and a useful benchmark for the development of next-generation membrane materials.

## Figures and Tables

**Table 1:** Reported bicarbonate and carbonate rejection performance of NF membranes under different operating conditions.

Reference	Membrane	Active Layer Material	MWCO (Da)	HCO_3_^−^ Rejection (%)	CO_3_^2−^ Rejection (%)
Rinberg et al. [34]	NF270	Polyamide TFC	300–400	40	~80–90
Zhu et al. [35]	NF90	Polyamide TFC	200	Not reported	>97
Zhu et al. [35]	NF270	Polyamide TFC	300–400	Not reported	>80
This Study	Toray RO	Polyamide TFC	Dense RO (MWCO not defined)	91	98
This study	Dow NF	Polyamide TFC	<200	60	95

## Data Availability

The datasets generated and/or analyzed during the current study are available from the author upon reasonable request.

## Supplementary Materials

The following supporting information can be downloaded at https://www.mdpi.com/article/10.3390/membranes16070248/s1, Section S1 presents the manufacturer specifications of the commercial reverse osmosis (RO) and nanofiltration (NF) membranes employed in this study. Section S2 describes the ionization of carboxylic groups and acetate as a function of pH, providing the theoretical framework for understanding membrane surface charge development and electrostatic rejection mechanisms. Section S3 presents the estimation of the osmotic pressure of the carbonate feed solutions and demonstrates that the applied transmembrane pressure exceeded the calculated osmotic pressure of all tested solutions, thereby ensuring a sufficient driving force for membrane separation. Table S1. Manufacturer specifications of the commercial membranes used in this study.
