# Supplementary material for: Selective Separation of Inorganic and Organic Carbonates in Aqueous Solutions by Reverse Osmosis (RO) and Nanofiltration (NF) Membranes
Source: Membranes (Basel). 2026 Jul 20;16(7):248. doi: 10.3390/membranes16070248 (PMC13414083; doi:10.3390/membranes16070248)
Supplement: Supplementary file 1 [file membranes-16-00248-s001.zip › membranes-4409181-supplementary.pdf]

Selective Separation of Inorganic and Organic Carbonates in Aqueous Solutions by Reverse Osmosis (RO) and Nanofiltration (NF) Membranes

Rahma Al Busaidi [1,2,*], Budoor Al Umairi [3], Zulfiqar Ahmad Rehan [3] and Mohammed Al-Abri [2,4,*]

1 Department of Physics, College of Science, Sultan Qaboos University, P.O. Box 33, Al-Khoud,
Muscat 123, Oman
2 Nanotechnology Research Center, Sultan Qaboos University, P.O. Box 33, Al-Khoud, Muscat 123, Oman
3 Department of Chemistry, College of Science, Sultan Qaboos University, P.O. Box 33, Al-Khoud, Muscat 123, Oman; bsss@squ.edu.om (B.A.U.); z.rehan@squ.edu.om (Z.A.R.)
4 Department of Petroleum and Chemical Engineering, Sultan Qaboos University, P.O. Box 33, Al-Khoud, Muscat 123, Oman
* Correspondence: rahmaalb@squ.edu.om (R.A.B.); alabri@squ.edu.om (M.A.-A.)

Section S1. Commercial Membrane Specifications

Table S1 summarizes the manufacturer-reported specifications of the Toray reverse osmosis (RO) membrane [1] and the DOW FilmTec nanofiltration (NF) [2] membrane employed in this study. These specifications provide a reference for interpreting membrane performance in carbonate separation experiments.

Table S1. Manufacturer specifications of the commercial membranes used in this study.

Parameter	Toray RO Membrane	DOW NF Membrane
Membrane Type	Reverse Osmosis (RO)	Nanofiltration (NF)
Manufacturer	Toray Industries, Japan	DuPont FilmTec, USA
Active Layer Material	Polyamide Thin-Film Composite (TFC)	Polyamide Thin-Film Composite (TFC)
Support Layer	Polysulfone/Polyester	Polysulfone/Polyester
Typical NaCl Rejection (%)	>99	40
Molecular Weight Cut-Off (MWCO)	Not typically defined for RO	200–400 Da
Operating pH Range	2–11	3–10
Supplier	Sterlitech Corporation, USA	Sterlitech Corporation, USA

Section S2. Ionization Of Carboxylic Groups and Acetate as A Function Of pH

Henderson-Hasselbach Equation is helpful to picture the acid's ionization [3], as follows:

$$\boldsymbol{HA \rightleftharpoons A^- + H^+} \tag{1}$$

The dissociation of the HA acid will occur at a certain rate characteristic of the particular acid. The acid dissociates into its component; the conjugate base of the acid and a hydrogen ion, but the ions rejoin again to form the original acid. A system is in equilibrium when the dissociation rate into ions equals the ion re-association rate. Strong acids will reach equi-librium when they are completely dissociated. A weak acid will have a lower percentage of molecules in a dissociated state. It will reach equilibrium at less than 100% ionization. The concentration of acid at equilibrium is called the acid dissociation constant, denoted by the

symbol Ka, given by the following equation:

$$\boldsymbol{Ka} = \frac{[H^+][A^-]}{[HA]} \tag{2}$$

A measure of Ka for a weak acid is given by its pKa, which is equivalent to the negative logarithm of ***Ka; pKa = –logKa and pH = –log[H⁺].***

Acid dissociation can simply be described by the mass balance equation as a sum of the dissociated anions, fraction of α_{A-}, and non-dissociated anions, fraction of α_{HA}. The non-dissociated fraction can be described as:

$$\boldsymbol{\alpha_{HA} = \frac{[HA]}{[HA]+[A^-]} = \alpha_0} \tag{3}$$

From the definition of ***Ka, [A–] = Ka [HA]/[H⁺]*** and hence,

$$\boldsymbol{\alpha_0} = \frac{[HA]}{HA] + (Ka \times \frac{[HA]}{[H^+]})} \tag{4}$$

$$\boldsymbol{\alpha_0} = \frac{[H^+]}{[H^+] + Ka} \tag{5}$$

The dissociated fraction can be described as:

$$\boldsymbol{\alpha_{A^-}} = \frac{[A^-]}{[HA]+[A^-]} = \boldsymbol{\alpha_1} \tag{6}$$

From the definition of ***Ka, [HA] = [H+] [A–]/Ka***, and so

$$\boldsymbol{\alpha_1} = \frac{Ka}{[H^+] + Ka} \tag{7}$$

Equations (5) and (7), used to describe the de/protonation of carboxylic groups on GO and ionization of carbonate as a function of pH.

Section S3. Osmotic Pressure Estimation of Carbonate Feed Solutions

The osmotic pressure of the carbonate feed solutions was estimated using the van't Hoff equation [4]

$$\boldsymbol{\pi} = \boldsymbol{i}\, \emptyset\, \boldsymbol{C}\, \boldsymbol{R}\, \boldsymbol{T} \tag{8}$$

where $\boldsymbol{\pi}$ is the osmotic pressure (bar), $\boldsymbol{i}$ is the stoichiometric van't Hoff factor representing the number of ions produced during solute dissociation, $\boldsymbol{\phi}$ is the osmotic coefficient (dimensionless), $\boldsymbol{C}$ is the molar concentration (mol L^{-1}), $\boldsymbol{R}$ is the universal gas constant (0.08314 L·bar·mol^{-1}·K^{-1}), and T is the absolute temperature (298 K). The osmotic coefficient accounts for non-ideal electrolyte behavior arising from ion–ion and ion–solvent interactions, which are not considered in the ideal van't Hoff equation. For dilute electrolyte solutions, ϕ is typically close to unity but decreases slightly with increasing ionic strength.

The stoichiometric van't Hoff factors were determined from the dissociation reactions of the carbonate salts in aqueous solution. Ammonium bicarbonate (NH_4HCO_3), potassium bicarbonate ($KHCO_3$), and sodium bicarbonate ($NaHCO_3$) dissociate into two ions and were assigned $\boldsymbol{i}$ = 2, whereas sodium carbonate (Na_2CO_3) and ammonium carbonate ($(NH_4)_2CO_3$) dissociate into three ions and were assigned $\boldsymbol{i}$ = 3.

$NH_4HCO_3 \rightarrow NH_4^+ + HCO_3^-$

$KHCO_3 \rightarrow K^+ + HCO_3^-$

$NaHCO_3 \rightarrow Na^+ + HCO_3^-$

$Na_2CO_3 \rightarrow 2Na^+ + CO_3^{2-}$

$(NH_4)_2CO_3 \rightarrow 2NH_4^+ + CO_3^{2-}$

Using a feed concentration of 0.1 M at 25 °C (298 K), the theoretical osmotic pressures calculated from the ideal form of the equation (ϕ = 1) were approximately 4.96 bar for NH_4HCO_3, $KHCO_3$, and $NaHCO_3$, and 7.44 bar for Na_2CO_3 and $(NH_4)_2CO_3$. The actual osmotic pressures are expected to be slightly lower due to non-ideal electrolyte effects (ϕ < 1). Nevertheless, all calculated osmotic pressures were substantially lower than the applied transmembrane pressure of 20 bar used during dead-end filtration experiments. Therefore, a positive net driving pressure was maintained throughout the study, indicating that permeation was not limited by osmotic pressure. Under these conditions, membrane performance was primarily governed by molecular sieving and electrostatic (Donnan) exclusion mechanisms.

References:

[1] Lenntech B.V. Toray Reverse Osmosis Membranes—Technical Specifications and Performance Data; Lenntech B.V.: Delfgauw, The Netherlands. Available online: https://www.lenntech.com/products/membrane/toray.htm (accessed on 15 June 2026).

[2] Sterlitech Corporation. Dow FilmTec Flat Sheet Membrane, NF90, PA-TFC, NF, 305 × 305 mm, 1/Pk; Sterlitech Corporation: Auburn, WA, USA. Available online: https://www.sterlitech.com/dow-nf90-nanofiltration-nf-membrane-305-x-305-mm-size.html (accessed on 15 June 2026).

[3] Po, H. N., & Senozan, N. M. (2001). The Henderson-Hasselbalch equation: its history and limitations. Journal of chemical education, 78(11), 1499.

[4] Kumar, T.; Jain, S. Solutions, pH, and Buffers. In Essential Laboratory Techniques and Biochemical Analysis; CRC Press: Boca Raton, FL, USA, 2025; pp. 29–58
